# Diversifying Description: Sweet Potato Science and International Agricultural Research after the Green Revolution

**DOI:** 10.1215/00021482-10474437

**Published:** 2023-08-01

**Authors:** Helen Anne Curry

**Affiliations:** History and Sociology Georgia Institute of Technology

## Abstract

The organization of sweet potato research across global regions began in earnest in the 1980s. Leading international institutions, notably CGIAR (Consultative Group on International Agricultural Research) recognized the potential for science-driven development of a “neglected” crop. Sweet potato was second only to potato in root crop cultivation worldwide and the top tuber in Asia yet had not been subject to the internationally coordinated research that its importance merited. In this paper, I explore how scientists involved in sweet potato research attempted to respond to the call for new international research and development efforts while avoiding the limitations of predecessor programs associated with the Green Revolution. I highlight the challenges inherent in this work by focusing on ambitions for—and challenges to—providing standardized information about samples of varieties used in research and entered into genebank collections. As scientists and institutions grappled with critiques of the top-down model of development, many sought to address these through more inclusive research practices. As I show, accommodating diversity in crops and among cultivators and cultures entailed costs that ultimately limited the longevity and effectiveness of some enterprises that sought to maximize inclusivity.

In March 1981, about one hundred agricultural researchers and administrators gathered in Taiwan for the First International Symposium on Sweet Potato. Their host institution, the Asian Vegetable Research and Development Center (AVRDC) was arguably the world-leading site for agronomic research on *Ipomoea batatas*. Yet despite the depth of knowledge and commitment to development of sweet potato on display at this meeting of experts from many nations, both scientists and crop sat on the margins of international agricultural research. Sweet potato experts had not received the same resources and opportunities as those working with maize, wheat, rice, and even white potatoes (*Solanum tuberosum*) in the post-war quest to resolve global food needs by driving up productivity. Meanwhile sweet potato ranked seventh among all crops globally in terms of metric tons of production per year and featured as nutritious, calorie-rich dietary mainstay across Asia and the Pacific. A high-yielding crop plant even without expensive inputs, it could be cultivated year-round and used both as fodder and food. These qualities suggested to the researchers gathered in 1981 that sweet potato was deserving of far more research attention than it received. It was, according to the Filipino vegetable breeder Ruben Villareal of AVRDC, “the world’s most underrated crop.”^1^

Sweet potato research fared better in subsequent years, a trajectory that must be understood in relation to broader trends in international agricultural research in the 1980s and 1990s. Institutions celebrated for fomenting a “Green Revolution” in the late 1960s came under intense scrutiny in the 1970s, charged especially with having advanced commercial, industrialized models of agriculture at the expense of subsistence cultivators.^2^ Administrators and funders of international research programs sought increasingly to incorporate neglected crops and cultivators into their efforts.^3^ Sweet potato was an obvious choice: already widely cultivated, very nutritious, potentially high yielding, suited to many ecologies, and yet not subject to the intensive research regimes that had led to productivity leaps in other crops.^4^ It was not a frictionless choice, however. Settling on sweet potato meant, among other concerns, dealing with an impressive diversity of biological forms and cultural practices that were less well documented within the international agricultural research system.^5^ “Improving” sweet potato therefore demanded the incorporation of new biological and agronomic expertise into established institutions. It also required knowledge of what sweet potato cultivators desired in their crop—which was clearly not just yield but myriad qualities associated with growing, storing, cooking, and more. Social scientists could help recover this knowledge; however, in the 1980s, their approaches remained on the fringes of international agricultural research.^6^

The history of sweet potato research therefore offers important insights into the history of international agricultural research and development. I join other scholars in looking beyond the well-worn narratives of a US-led and geopolitically motivated Green Revolution in wheat and rice to see the other actors and settings—and crops—implicated in late-twentieth century agricultural transformations.^7^ My analysis foregrounds early efforts from within established research centers to overcome limitations of the breeder-driven, commodity grain–focused approach that predominated through the 1970s. In ramping up sweet potato research, international research programs attempted to “improve” a heterogeneous subsistence crop of minor importance in global trade; in recognizing the need to better serve subsistence cultivators hailing from diverse cultures and climates, they increasingly relied on sociologists and anthropologists to make these cultivators’ needs known.

These were not shifts easily made. As my analysis reveals, the effort to internationalize sweet potato research while avoiding the limitations of predecessor programs associated with the so-called Green Revolution encouraged new approaches in crop science. I focus on the task of providing standardized information about samples of varieties that were used in research and entered genebank collections. Within the international agricultural research consortium CGIAR (Consultative Group on International Agricultural Research, introduced below), “minimal” crop descriptor lists focusing on key traits and standard ways of recording these were envisioned from the early 1970s as means of facilitating the exchange, evaluation, and conservation of breeding materials across its institutional network and beyond. Sweet potatoes initially proved no exception to this pattern. However, in the 1980s, the costs of minimalism and the impossibilities of universalization drove crop descriptor development and other documentation efforts towards more inclusive and flexible approaches. As I recount here, sweet potato researchers led the way towards new maximalist interpretations.^8^

The history of efforts to describe sweet potato in ways that made research accessible to and encompassing of a greater number of actors highlights the challenges facing researchers working in international institutions in the decades after the initial celebration-turned-condemnation of the Green Revolution. As scientists and institutions grappled with critiques of the top-down model of development, many sought to address these through more inclusive research practices. One strategy was to diversify research, for example by bringing in new crop species and academic disciplines, and, relatedly, to more fully recognize the biological and cultural diversity seen on farms. As I show, accommodating diversity in crops and among cultivators and cultures came with costs—ones that ultimately limited the longevity and effectiveness of some enterprises that sought to maximize inclusivity.

## International agricultural research, global geopolitics, and the side-lining of sweet potato

As many historians have recounted, the 1960s and 70s saw tremendous institution building in international agricultural research for development, including the creation of the influential Consultative Group on International Agricultural Research (CGIAR).^9^ New institutions, often building on substantial local experience and historic national investments in agricultural science, generated opportunities for more researchers to engage in a wider range of research activities linked to international agricultural development. But not all research communities benefitted equally. In comparison to major commodity grains like wheat, maize, and rice, roots and tubers including sweet potato received comparatively short shift within expanding research infrastructures. As I describe here, the first task in organizing an influential cross-border research community centered on sweet potato lay in overcoming this marginality.

The rapid institution building in international agricultural research in the 1970s was motivated especially by evidence that investments in centralized plant breeding research (combined with greater availability of fertilizers, irrigation, and credit schemes) produced significant increases in the production of staple grains. Two institutions were particularly influential in producing this understanding through their launch of high-yielding varieties of wheat and rice in the 1960s: the International Center for Maize and Wheat Improvement (Centro Internacional de Mejoramiento de Maíz y Trigo; CIMMYT) in Mexico and the International Rice Research Institute (IRRI) in the Philippines. At these centers, private philanthropies (namely, the Rockefeller and Ford Foundations) and host country dollars sustained researchers from many countries in the pursuit of greater agricultural productivity. In many historical accounts, it was their wheat and rice that proved central to the so-called Green Revolution of the late 1960s and early 1970s.^10^ Although the costs and benefits of the Green Revolution for farmers and communities in Asia, Latin America, and other regions of the Global South are still actively debated, at least one outcome is beyond dispute.^11^ Bumper harvests inspired faith that further such harvests could be created by expanding infrastructure for agricultural research targeted to the needs of developing nations—including by building new scientific centers on the model of CIMMYT and IRRI.^12^

From 1971 onward, the coordination of international aid money to sustain four existing international agricultural research centers that had been created by the Rockefeller and Ford Foundations, and to develop new such centers, took place via CGIAR. A scientific advisory board, the Technical Advisory Committee, guided CGIAR members in deciding what research to prioritize and whom to enlist in doing that research. From its first four centers, which included the grain commodity–focused CIMMYT and IRRI as well as two tropical crop research centers with more diversified research portfolios,^13^ CGIAR sprawled outwards to encompass existing research enterprises and launch new ones. By the end of the 1980s it had added a potato institute in Peru (discussed below), a rice facility in West Africa, centers for semi-arid agriculture in India and drylands in Syria, two livestock research operations in Africa, as well as several policy-oriented agencies in the US and Europe.^14^

A conspicuous absence among the many research responsibilities divvyed up among these institutions was vegetables. Of the institutes attentive to crops, the commodity centers delivered on corn, rice, wheat, white potatoes; the tropical centers on forage grasses, food legumes (beans, cowpeas, soybeans), and plant starches (especially cassava); and the arid and semi-arid centers on grains (especially millets) and food legumes. Within the 1970s CGIAR vision of feeding the world—which is to say, in the eyes of the predominant international agenda-setting body—there was no room for cabbages, carrots, and cucumbers.

It had not been for lack of opportunity. Even as the CGIAR network of research institutions came together in the early 1970s, the Asian Development Bank, US Agency for International Development, and several national governments (Japan, Korea, Philippines, Taiwan, Thailand, and Vietnam) had agreed to found the Asian Vegetable Research and Development Center (AVRDC). Originating in existing institutional infrastructure for crop science in Taiwan, AVRDC was to focus on crops that were richer in protein, vitamins, and minerals than staple grains and which, because of high demand, would “give promise of higher income, as well as improved nutrition, for the Asian small farmer who often depends almost entirely on rice for his income and food.”^15^ By the time of its first annual report in 1974, AVRDC claimed six research targets: mung beans, soybeans, tomatoes, sweet potatoes, white potatoes, and Chinese cabbages.^16^

Under the leadership of Director General Robert Chandler, an American agronomist who was a Rockefeller Foundation employee and former director of IRRI, AVRDC approached CGIAR in 1972 to ask for inclusion in its expanding network of institutions; the sought-after affiliation would expand its access to the global donor community.^17^ CGIAR advisors had doubts, however. Some worried that vegetables were “luxury crops” and others that research on these would augment export production rather than sustain local dietary needs.^18^ At one crucial decision-making meeting, advisors “pointed out that the problems of calorie and protein nutrition had not yet been solved, and that these should continue to be rated higher than mineral or vitamin deficiencies for some years to come.”^19^ In other words, in a hungry world, vegetables just didn’t merit CGIAR attention. Even as it allayed these concerns by making a case for greater attention to the quality of diets, AVRDC confronted a very different obstacle to its activities, one that would ultimately do more to thwart its inclusion in CGIAR. In October 1971, the United Nations general assembly voted to admit the People’s Republic of China—and to expel the Republic of China (Taiwan). By 1975, Japan, Thailand, and the Philippines had all severed diplomatic ties with Taiwan, forcing the Taiwanese government to step up its support of an organization now entangled with its contested claims to national sovereignty. Although AVRDC was repeatedly brought up as a candidate for admission to CGIAR in subsequent years, the political obstacles proved insurmountable.^20^

If AVRDC remained on the margins, not all its priority vegetables were equally marginal. In privileging both sweet potatoes and white (also sometimes called Irish) potatoes over other vegetables, the AVRDC had produced a priority list that overlapped with established CGIAR research areas. The tropical agriculture center in Ibadan (IITA) had from its launch in 1967 sustained work on several starchy tubers important in African diets. In 1971 CGIAR leadership confirmed their intention that this center should have “world wide responsibilities covering all climatic regions” for yams, taro, and sweet potatoes.^21^ By 1983, its sweet potato research portfolio included screening lines introduced from across Africa, Asia, and the Americas for resistance to pests and diseases, producing clones of resistant types for distribution, developing new populations through selective breeding, and developing tools like tissue culture techniques in order to safely share breeding materials across institutions without transmitting viruses.^22^

The other major CGIAR site for tuber research was located in Peru. In 1972, CGIAR had begun providing funds to the International Potato Center (Centro Internacional de la Papa, CIP).^23^ The origins of this institution lay in the “North Carolina Mission to Peru,” an agricultural assistance program begun in 1954 and funded by the US Agency for International Development. (Its first Chief of Mission was Ralph Cummings, who would go on to lead IRRI and hold other high-level positions in CGIAR institutions.) The “Mission” placed North Carolina State University staff as technical experts in Peruvian institutions. In 1966, resources from this program, which had served multiple facets of agricultural research and production, were allocated to create a centralized national research program centered on white potato. The program had strong backing from within Peru: in 1967 the President of Peru, Juan Velasco Alvarado, issued a decree establishing an International Potato Center. This institution was ostensibly to follow the model of IRRI and CIMMYT—that is, it would be a global research institution founded in a strong national program and focused on a crop commodity.^24^

Funding remained in question, however. CIP’s founding director, the North Carolina State University professor Richard L. Sawyer, sought funds from European and North American governments and philanthropies, only to find himself “in competition with 15 to 20 Centers which various groups were trying to get started due to the successes of the Corn Wheat Institute [CIMMYT] and the Rice Institute [IRRI].”^25^ The nascent potato center emerged victorious in this scramble. As one of the first new centers to be incorporated formally into CGIAR, the International Potato Center had secured long-term funding by 1972 and took on the project of expanding production of the white potato, chiefly on the premise that its potential as a nutritious, high yielding food in the lowland tropics had yet to be fully realized.^26^

The programs at IITA and CIP indicated high-level recognition in CGIAR of the importance of tubers to global diets, albeit chiefly in their role as starchy staples rather than nutrient-rich vegetables.^27^ The comparative marginality of roots and tubers among CGIAR initiatives—and the prioritization of white potato over sweet potato, taro, cassava, and other tubers—nonetheless underscored the limits of the CGIAR vision of agricultural modernization. Its initial focus on increasing the productivity of staple crops that were also commodities important to developed world industries and economies spoke to the geographical origins of its leading scientists and administrators and the geopolitical inclinations of its leading funders.^28^ In a summation of the state of research on starchy roots and tubers in 1971, the UN Food and Agriculture Organization noted that “with the exception of white potatoes these crops are grown and consumed almost entirely in developing countries, and hardly enter into world trade. They have therefore received little attention in research programmes in developed countries.” Its advocacy to CGIAR for a “carefully planned programme” of research on these crops, which could “transform the diet in some of the worst areas of malnutrition where it is difficult to grow cereals” produced little effect on the overall CGIAR research agenda—and by extension, on the larger sphere of international agricultural research for development— in CGIAR’s early years.^29^

This context explains why sweet potato, a crop of major dietary significance in regions in targeted for agricultural development aid, remained at the margins of international agricultural research initiatives in the 1980s. Within the expanding CGIAR, work on sweet potato was constrained to the International Institute for Tropical Agriculture in Ibadan, where it competed for resources not only with other tropical tubers but also the broader portfolio of crop and livestock research assigned to the center.^30^ Meanwhile, sweet potato researchers at AVRDC, situated in Asia where more than 80 percent of the world’s sweet potatoes were grown, were distanced from the core of funding for international agricultural research by dint of their association with the “less important” vegetables and the geopolitical side-lining of Taiwan.

## Internationalizing sweet potato research in the 1980s

In 1985, the geography of internationally funded sweet potato research shifted. Following two influential top-level studies at CGIAR, one indicating that “sweet potatoes were considerably underfunded” in comparison to their acknowledged importance in many regional food systems and a second that the “most successful” of the CGIAR research centers were those focused on specific commodities, the International Potato Center in Peru began to shift some of its research funds from *Solanum tuberosum* to *Ipomoea batatas*.^31^ Whereas earlier decisions had scattered the responsibility for maintaining global collections of sweet potato germplasm across several institutions, this responsibility was soon transferred exclusively to CIP in Peru. As I describe here, CGIAR’s bid to centralize sweet potato research in the international research system, which included bringing together collections of tubers and seeds from multiple sites, generated new challenges. As scientists assigned to the collection attempted to whittle it down to a manageable size, they found themselves in need of more data—and a significantly expanded vocabulary.

The initial move of CIP into sweet potatoes took place as CGIAR leadership, confronted by worrying shortfalls in funding, grappled with what the priorities for its international agricultural research centers should be. After a decade of institutional expansion and steady growth in donor support, funding stagnated in the early 1980s and international research centers’ budgets plateaued.^32^ In 1983, CGIAR’s scientific advisors reassessed the overarching priorities of CGIAR and the mandates of the individual centers. The review of priorities, prepared by the plant physiologist and CGIAR Technical Advisory Committee member Lloyd T. Evans, noted three crops that probably deserved more attention given their importance in global diets—barley, sweet potatoes, and plantains. Lloyd further reminded his colleagues that in terms of the number of low-income countries growing significant acreage and the percent of calories derived from the crop in those countries, “sweet potatoes are at least as important as potatoes.” He therefore concluded that, “based on present production and trends, research on the potato within the CGIAR is overfunded, certainly in relation to that on cassava and sweet potatoes.”^33^ Meanwhile the review of centers’ mandates, and their successes in achieving those mandates, observed that although CIP’s mandate “allowed it to work on the potato and other tuberous roots” (emphasis in original), it had “on its own… decided to limit its activities [to] the potato.”^34^ In short, the assessments suggested too much CGIAR spending on potato, too little on sweet potato, and highlighted a center perfectly positioned to redress the balance.

The message clearly made its way to CIP leadership: its Board of Trustees decided to allocate special project funding to sweet potatoes in 1984.^35^ It earliest sweet potato research activities—collecting and sharing hundreds of Latin American strains of sweet potato—point to justifications beyond institutional survival that explain its entry into a territory still explicitly marked out for a different CGIAR institution.^36^ Because *Ipomoea batatas* had originated in the Americas, diverse local populations and wild relatives were found across several Central and South American countries. Meanwhile, the substantive collections of sweet potatoes for use in breeding and research were held outside the region. When the International Board for Plant Genetic Resources—a CGIAR institution tasked with coordinating the conservation and use of crop genetic resources across international institutions—first turned its attention to sweet potatoes around 1980, it recommended field collecting in Guatemala, Ecuador, Colombia, and Peru to secure samples from key sites of genetic diversity in Latin America. By comparison, samples reflecting the diversity of the crop across Asia and the Pacific were to be obtained by aggregating existing research collections. The International Board had then assigned responsibility for maintaining these collections to institutions in the United States, Africa, and Asia.^37^

In addition to its being ideally located within a region of critical extant sweet potato diversity, CIP also possessed crucial technical expertise. The transfer of sweet potatoes’ fleshy tubers—the typical means of exchanging germplasm in this clonally propagated crop—easily resulted in the transfer of viruses if care were not taken to ensure virus-free stocks. For a field collection of sweet potatoes an introduced virus could entail serious losses. At AVRDC, the procedures for preventing disease introduction via imported samples included cultivating newly arrived material in quarantine screenhouses impervious to the pests known to transmit viruses, insecticide and fungicide treatment of all materials, acquisition of meristem tissue cultures which would be used to produce new tubers, and finally destruction of all the original plants and tubers.^38^ CIP scientists and technicians already had expertise in maintaining and circulating virus-free potato samples, following similar procedures.^39^

In short, as the demand for sweet potatoes for research and breeding accelerated, including samples of landraces and wild relatives from Latin America, CIP was ideally positioned, geographically and technically, to become the hub for the circulation of these materials too.

As CIP moved more assertively into sweet potato science from 1985 onward, effectively becoming a two-commodity center, it drew on the established expertise and resources of both national and international research organizations. Its collaborators included scientists based at international operations such as IITA and AVRDC and state institutions such as North Carolina State University and various national agricultural research programs.^40^ Its collections came from its own (internationally organized) initiatives, but also donations. Of the 3,846 samples in the collection by 1988, 766 were donated from the collection of the Peruvian agronomist Romulo del Carpio, 565 from a University of Ayacucho collection assembled by Peruvian scientist Carlos Arbizu, with another 300 some samples coming from six further Peruvian institutions as well as collections in Costa Rica, Dominican Republic, China, Japan, and the United States.^41^ It also depended on existing infrastructure that sustained these collaborations and exchanges, from standardized approaches to identification to agreed disease screening and quarantine procedures.

Among the infrastructural elements considered crucial to the development of a global sweet potato genebank collection—a repository of breeding materials that would in turn be the foundation of CIP’s contribution to sweet potato research and improvement worldwide—were crop descriptor lists. These lists dictated the basic identifying information and characteristics of a sample to be documented by researchers (“crop descriptors”) and provided the terms in which they were to be recorded (“descriptor values").^42^ A vision of culturally, linguistically, and disciplinarily diverse researchers generating knowledge about plant breeding materials that could be used by anyone, anywhere and of a system of germplasm repositories made accessible through computerized databases encouraged a particular focus on “minimal” descriptor lists. Developers imagined that a shared, spartan vocabulary would facilitate genebank curation, create frictionless communication among researchers from diverse disciplines and institutions, and aid in the exchange of breeding materials especially across international borders.^43^

**Figure 1:**
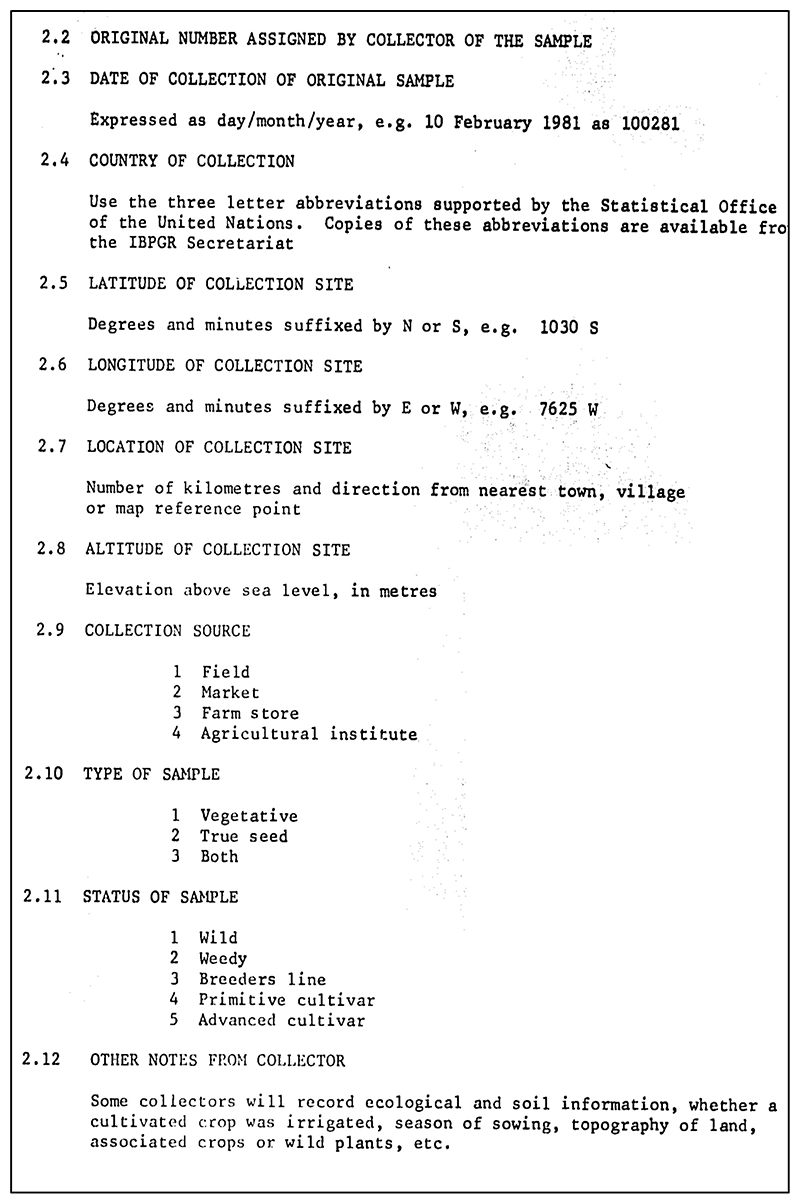
A subset of the crop descriptors to be used by plant collectors when obtaining samples of sweet potato varieties in the field, as set out by an international expert meeting in 1980. From IBPGR, *Genetic Resources of Sweet Potato* (Rome: IBPGR Secretariat, 1981), 21. Republished by permission of Bioversity International.

**Figure 2:**
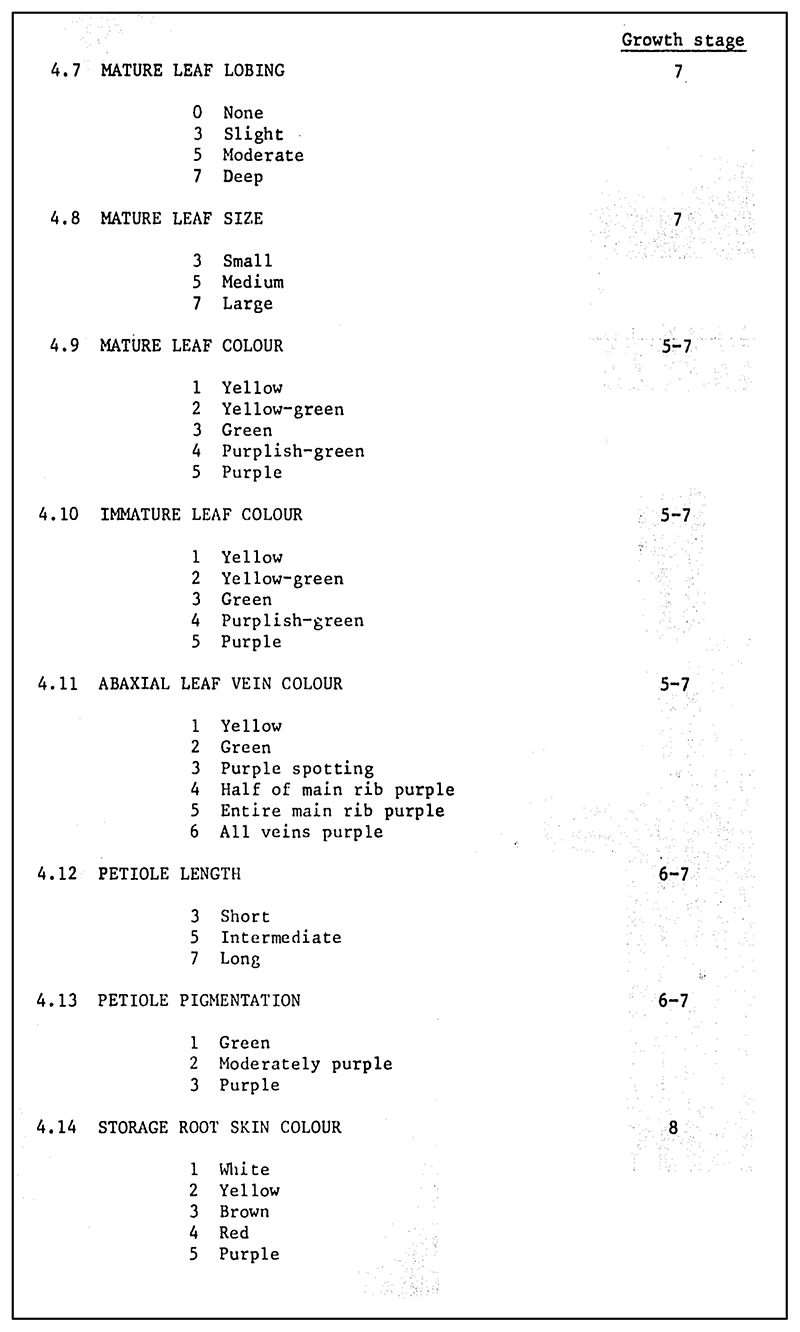
A subset of the crop descriptors to be used by plant breeders and other researchers when evaluating sweet potato plants, especially those associated with genebanked samples. From IBPGR, *Genetic Resources of Sweet Potato* (Rome: IBPGR Secretariat, 1981), 23. Republished by permission of Bioversity International.

As the CGIAR entity responsible for the management, conservation, and exchange of crop genetic materials, the International Board for Plant Genetic Resources was tasked with elaborating descriptor lists for all crops of sufficiently wide interest within the CGIAR network. It had first turned its attention to sweet potato in 1980, motivated by escalating concerns about the potential loss of genetic diversity in sweet potato varieties and the need to systematize collections already held by leading institutions. The International Board published its first-ever descriptor list for sweet potato in 1981. Experts from Australia, Nigeria, Taiwan, and the United States had convened to agree on an adequate set of crop descriptors and descriptor values.^44^ Their eventual agreed list covered 116 potential data points, from latitude and longitude of original collection to petal shape to keeping quality of tubers; a full 52 descriptors charted a sample’s known responses to specific pests and diseases.^45^ To take just two examples, a collector obtaining a new sample would record descriptor 2.9, Collection Source, as: 1. Field, 2. Market, 3. Farm Store, or 4. Agricultural Institute; a breeder running field studies would record descriptor 4.14, Storage Root Skin Color, as either: 1. White, 2. Yellow, 3. Brown, 4. Red, or 5. Purple; and so on (see Figures 1 and 2).

This 1981 list was a crucial tool for the Peruvian agronomist Zósimo Huamán as he began working with the samples of sweet potato and its wild relatives arriving at CIP from collecting missions and other institutions in the mid-1980s. Huamán had been hired by CIP in 1976 and placed in charge of the center’s world collection of (white) potato. At the time, he had been a newcomer to neither CIP nor to potato collections. Educated in agronomy at the Universidad Nacional Agraria La Molina, Huamán had helped to organize the Peruvian national potato collection at the La Molina experiment station while still a student and worked with the North Carolina Mission to Peru after his graduation in 1969. He subsequently joined potato collecting trips across Peru and Bolivia, experiences that led him to study potato genetic diversity under the British scientist Jack Hawkes. Between 1972 and 1975, he had conducted most of his doctoral research on potato relatives in the fields and greenhouses at CIP.^46^

In 1981, after five years managing CIP’s potato collection, Huáman was well familiar with the demands that organizing its new world sweet potato collection would entail. He needed to sort incoming materials from existing national sweet potato collections and more recent explorations, verify or determine the identity of each, cull obvious duplicates, systematically document remaining materials, register everything in a computer database, determine which materials to keep alive as living plants in the field genebank and what to attempt to store as seed or tissue in the *ex situ* seed genebank, and keep those genebank accessions safe and genetically stable for years to come.^47^

Following widely shared thinking in genebank curation, Huamán deemed the value of the materials in the CIP sweet potato collection to be dependent on the quality and availability of the data about them. He and his colleagues therefore aimed to compile all basic identifying information (so-called passport data), details about the circumstances of original field collection, morphological characterization (basic observable qualities of tuber and plant) and preliminary evaluation data (the performance of a particular sample in field evaluations) using consistent descriptors and ultimately to enter these into a computer database (though this was still in development).^48^ Consistent characterization and evaluation of samples would facilitate local management as well as wider access and circulation. Huamán was especially concerned about the extent of duplication in his collection—that is, instances in which two or more samples originated in the same field population (or even the same original collection). Duplicate samples meant wasted resources, especially for a crop species that could not be kept as seed but needed to be kept in near-continuous cultivation in the field as was the case for sweet potato at the time. Carefully attending to descriptors would produce information valuable for identifying potential duplicates.^49^

Huamán quickly encountered problems in his application of the descriptor list published in 1981 following the international meeting of sweet potato researchers: it didn’t match up to the diversity he saw in his collection.^50^ Nor was he the only researcher to encounter this problem. An account from AVRDC in Taiwan noted that researchers had “closely followed” the agreed descriptor list when characterizing the sweet potato collection but then described how they had modified these “by adding new categories and/or by including additional characters to improve characterization efficiency.” The report also noted that it had proved impossible to record the requested floral characteristics for many samples because not all samples flowered in the ecological setting at AVRDC.^51^ Meanwhile in Papua New Guinea a researcher dispatched to collect and characterize the incredible diversity of sweet potato there relied on an abbreviated list of 27 descriptors from the longer list and made modifications within these to accommodate her samples.^52^

Huamán felt that the variation he and others observed in the field had “made [it] necessary to develop a more comprehensive list" of sweet potato descriptors, not just to address his local needs but also to guide international research.^53^ For his initial characterization of the sweet potatoes at CIP, he had relied on an expanded list of descriptors that he compiled from the “official” list produced by the International Board for Plant Genetic Resources plus the modifications made by the researcher in Papua New Guinea and additional descriptors proposed by researchers charting sweet potato diversity on Fiji. He and other CIP researchers considered a still “more comprehensive list” to be of “major importance” to their work, not least to keep on top of the inevitable arrival of duplicates. First published in 1988, this expanded list included: “most” of the morphological and evaluation characteristics in the original list; modifications to the list proposed based on the work in Fiji and Papua New Guinea; and further characters used to describe varieties in scientific publications originating in the Americas, Southeast Asia, and the Pacific.^54^

**Figure 3:**
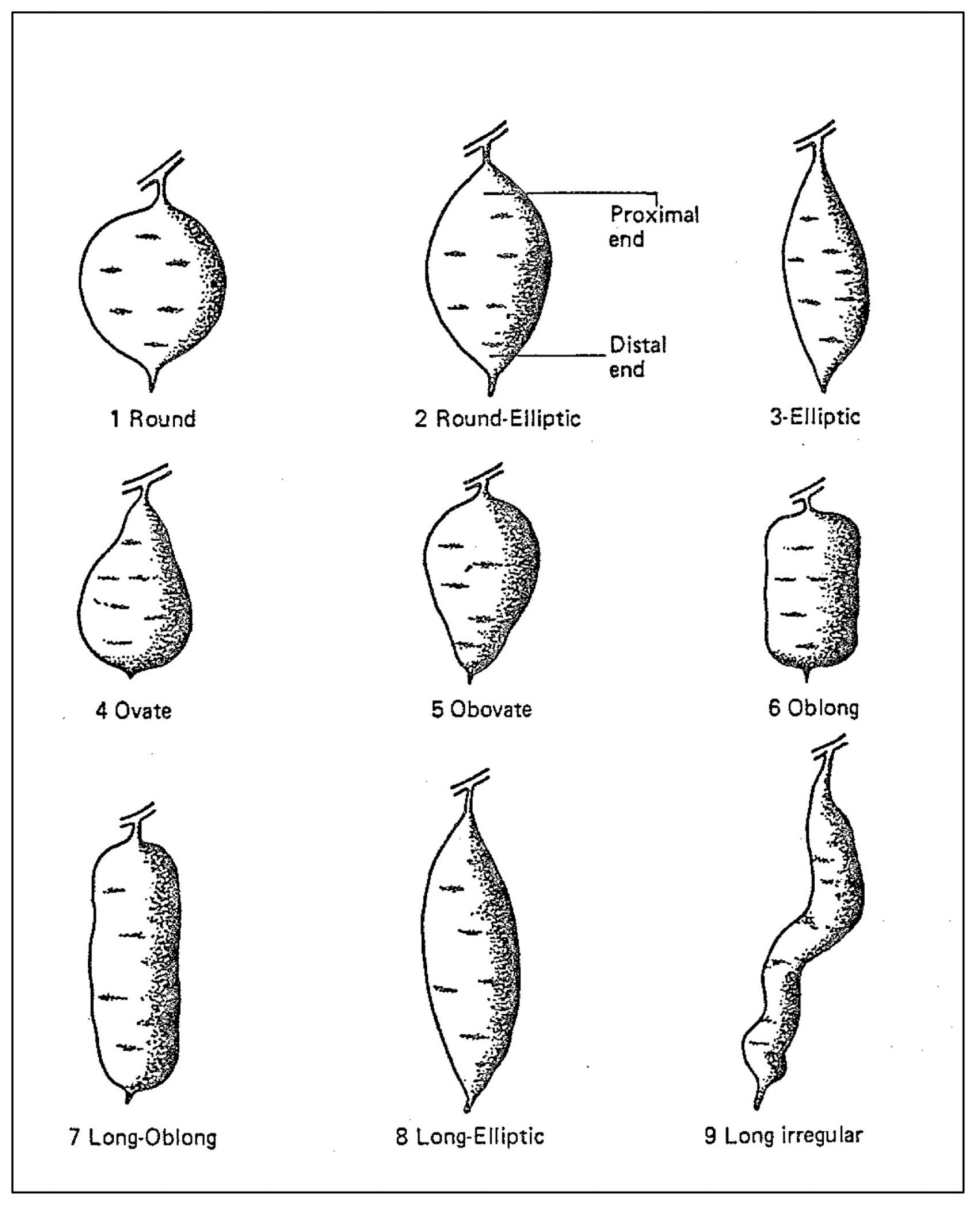
A diagram indicates the expected form associated with different descriptors for “storage root shape” in sweet potatoes. From Zosimo Huamán, “Descriptors for the Characterization and Evaluation of Sweet Potato Genetic Resources,” in *Exploration, Maintenance, and Utilization of Sweet Potato Genetic Resources: Report of the First Sweet Potato Planning Conference 1987* (Lima, Peru: CIP, 1988), 340. Republished by permission of the International Potato Center.

The result was a list that was modestly expanded in terms of qualities targeted by descriptors—for example, 33 descriptors for morphological characterization compared to the original 26, 10 preliminary evaluation descriptors compared to the original 5, and so on. Characteristics newly targeted for description included the shape of the sweet potato tuber. The options for tracking this trait on Huamán's updated list included 1. round, 2. round-elliptic, 3. elliptic, 4. ovate, 5. obovate, 6. oblong, 7. long-oblong, 8. long-elliptic, and 9. long-irregular, with shapes coded in the database according to the assigned number and researchers provided with a set of drawings to guide their classification (see Figure 3). Further down the list an entirely new subset of evaluation characteristics charted the qualities of the sweet potato tubers when boiled: their consistency, texture, sweetness, and coloration considered undesirable. In other words, the expanded list considered some of eaters' concerns alongside those of breeders and farmers.^55^

If the number of additional traits sought remained modest, the range of “descriptor states” offered for traits in many cases underwent dramatic expansion. Clearly the original list had not adequately encompassed the range in even the most obvious traits likely to be encountered by researchers at field sites around the world.

Consider the assessment of the sweet potato tuber's skin color (“storage root skin color”). In the original agreed descriptor list, this could be 1. white, 2. yellow, 3. brown, 4. red, or 5. purple.^56^ By comparison a researcher using the expanded 1988 list would have the option of recording both a “predominant” and a “secondary color” for the potato's skin and could choose for each: 1. white, 2. cream, 3. yellow, 4. orange, 5. brown, 6. pink, 7. red, 8. purple-red, or 9. dark purple. A researcher's options for noting the color of the potato's inner flesh similarly multiplied, from four hues to nine plus an additional list of nine possible patterns for anthocyanin pigmentation distribution.^57^

For Huamán, having a wider range of possible descriptions was not just about adequately capturing the phenotypic and other diversity seen in sweet potato, although this was certainly a concern. It was also a means of disciplining scientists. He saw descriptors and detailed information on how to record these as a means of eliminating the “subjectivity of the evaluators” from the process of characterizing and evaluating genebank samples. With clear instructions on the conditions in which to grow and observe samples, which traits to measure and observe, and the exact words to use in documenting these observations, plants could be precisely specified in a database and duplicates in the collection would become obvious.^58^ In this way, his revisions adhered to the original goal set out for crop descriptors: managing the diversity of researchers as well as crop plants. Making the sweet potato descriptor list more accommodating to both kinds of diversity was a means of expanding rather than ceding control.

As lengthy as it had become by 1988, the sweet potato descriptor list had not yet reached its full extent. With Huamán leading, the International Board for Plant Genetic Resources collaborated with CIP and AVRDC on the publication of a new official list that would replace the one published in 1981. Huamán had especially wanted a better tool for local collection management, but others were more concerned with international coordination. When researchers gathered in China to discuss a proposed Sweet Potato Genetic Resources Network that would link institutions and researchers across Asian countries, they were advised by a researcher from the International Board for Plant Genetic Resources that such a network would depend on all members having “ready access to all the information on all accessions [genebank samples] conserved by the entire network.” They would have to “give highest priority to the documentation of their sweet potato germplasm collections” and agree to “strictly follow” a set of agreed descriptors and descriptor states.^59^ This perspective on the purpose of descriptors adhered closely to the concepts that had driven their earliest development—and that also informed the production of a new official descriptor list with Huamán at the helm. As its preface described, the list was intended as a “universally understood ‘language’ for all plant genetic resources data” linked to sweet potato whose adoption by researchers would “produce a rapid, reliable and efficient means for information storage, retrieval and communication.”^60^

When the CGIAR network initially pursued the production of crop descriptor lists in the 1970s (chiefly through the International Board for Plant Genetic Resources), the emphasis had been on “minimal” lists. Researchers were busy and short on funds, the thinking went, and therefore should be asked to provide the bare minimum of information necessary to facilitate the exchange of seeds and knowledge. In 1980, an advisory panel to the International Board had urged against “over-elaborate descriptor lists,” calling these “self-defeating” and recommending instead that lists be kept “as short as possible” by focusing on the institutional identifiers and “basic botanical characters.”^61^ By comparison, in 1992, descriptor lists were viewed not as minimal but as maximal and celebrated as providing “the widest number of descriptors that will assist with the characterization of the crop.”^62^ The new sweet potato descriptor list, published in 1991, exemplified this more comprehensive approach. It featured dozens of additional characters and data points added over and above what had already been introduced by Huamán in his 1988 list.^63^

Zósimo Huamán’s experiences organizing the CIP sweet potato genebank provide a partial explanation for this shift: the condensed lists considered most suitable for computerized data management in the 1980s were less suitable for the plants and projects that researchers actually faced in the field and genebank. This was no doubt especially true where international researchers sought to encompass crops like sweet potatoes, whose biological constitution had been less subject to intensive research and development and which remained largely unconditioned by industrial production. Conducting international research might require a common language, but that language had to be flexible and expansive enough to encompass a wide range of experiences.

## Local knowledge, farmer experiences, and the limits of description

But just how flexible could descriptor lists, and the databases they were designed for, become? Social science researchers moving into international agricultural research soon pushed them to the limits. As I show here, anthropologists working directly with sweet potato cultivators, often as a route to exploring modes of agricultural research more responsive to farmers’ needs and expectations, found that tracking farmers’ knowledge about sweet potato varieties necessitated renewed negotiation of the terms and tools of crop description.

Even as CGIAR took shape, motivated by a vision of extending the Green Revolution wheat and rice harvests of the 1960s to other sites and other crops, its dominant model of agricultural transformation—and the research that would underpin this transformation—was under increasingly intense scrutiny. Critiques of the Green Revolution induced a re-examination of not only research priorities (like what crop species to study) but also research methods.^64^ In the mid-1970s, responding to concerns that a relentless focus on driving up yields of commodity grains ignored farmers’ actual needs, several CGIAR institutes started programs that encompassed a “farming systems approach.”^65^ This was an approach to agricultural research inspired chiefly by “a problem widely identified across the developing world,” as one early advocate of farming systems research summarized, that “technologies recommended as a result of agricultural research investment were, in general, inappropriate to the priorities and circumstances of small farmers.”^66^

Researchers who engaged in the study of farming systems sought to chart the operations and decision-making patterns of farmers and their households, in the hopes that this knowledge could underpin more relevant interventions. This work of necessity demanded the engagement of a wider range of disciplinary expertise than was encompassed in most natural science–dominated crop improvement programs. Anthropologists, sociologists, economists, and other social scientists were indispensable to the surveys and assessments that formed the foundation of farming systems and related farmer-centered approaches to agricultural research.^67^ So, too, were farmers and their families, as research increasingly validated knowledge characterized (not unproblematically) as “local” or “indigenous,” including within CGIAR.^68^

One of the most influential models for farming systems research emerged from studies conducted at CIP in the 1980s. The ‘farmer-back-to-farmer’ approach, inspired by an interdisciplinary effort to develop post-harvest storage and processing technologies, emphasized that research needed to begin and end with the farmer in order to be effective.^69^ The anthropologist on the team and co-author of the farmer-back-to-farmer concept was Robert Rhoades. A postdoc at the time, Rhoades’ position at CIP had been made possible via a Rockefeller Foundation initiative that aimed to increase social sciences fieldwork in CGIAR. Reportedly, CIP had been the first center to request an anthropologist through the program.^70^ Rhoades would go on to become one of the first full-time anthropologists to be supported by core CGIAR funding, as well as an effective champion of research that facilitated the participation of farmers in agricultural research and technology development.^71^ Nearly a decade after his arrival at CIP in Peru, Rhoades moved to the Philippines to launch the CIP program Users' Perspective with Agricultural Research and Development (UPWARD). This Asian sweet potato and potato research network was grounded in the farmer-back-to-farmer model and aimed “to support user sensitive agricultural research” by focusing on farm-level experiences.^72^ UPWARD was not a top-down program organized around technical exchange or technology transfer but instead a heterogenous network of researchers and institutions that assembled around a common commitment to farmer-centered projects. As Rhoades later described, it was a space “where anyone that was interested in building the user’s perspective into the agricultural research process could [get] small amounts of funding.”^73^

Sweet potato cultivation was an unquestionably good object of farmer-centered participatory research. It was globally significant in terms of providing calories, yet not because of international commodity markets. Researchers in the 1980s considered it “an emergency crop” and “a crop of the smallest and poorest farmers.”^74^ Sweet potatoes were typically cultivated on marginal lands without costly commercial inputs like fertilizers, often with the significant involvement of women. Sweet potato growers deployed a variety of cropping systems, and in many cases grew multiple varieties on a single farm. The crop also had a range of uses, from starchy vegetable to confectionary production to leafy greens to animal feed, depending on context. Sweet potatoes therefore provided ample opportunities for researchers to delve into local circumstances and needs and to address the needs of diverse cultivators including women. Because farmers chose varieties based on taste and texture (for example) rather than yield, breeding programs would have to target farmers’ preferences to create varieties those farmers would actively choose to plant.^75^

Formally launched in 1990 with support from the Dutch government, the UPWARD program positioned itself in explicit contrast to the wheat and rice programs of the Green Revolution. As the CIP anthropologist Gordon Prain (Rhoades’ eventual successor as the UPWARD coordinator) described, the failure of the Green Revolution to offer benefits to many socially and economically marginalized farmers had catalyzed a further revolution, this one “more of attitudes than technology” and positioned to “turn upside down the conventional perspectives, philosophies, and methods of the agricultural development community.” UPWARD, like other farmer-centered initiatives with which its organizers and participants identified, would work more closely with households, women especially, and would tackle problems beyond baseline crop productivity to consider distribution, consumption, and resource management. Above all UPWARD projects would demonstrate “a core of respect for indigenous knowledge and a desire to work with and build upon local agricultural practices.” For those observers worried that a commodity focus (i.e., potato production) was incompatible with farmer-centered participatory research, Prain emphasized the suitability of sweet potato science for establishing a new model of agricultural research for development. It was, he insisted, a low-input crop of marginal lands “frequently identified as a good candidate for diversifying and sustainably intensifying crop production systems.”^76^

**Figure 4:**
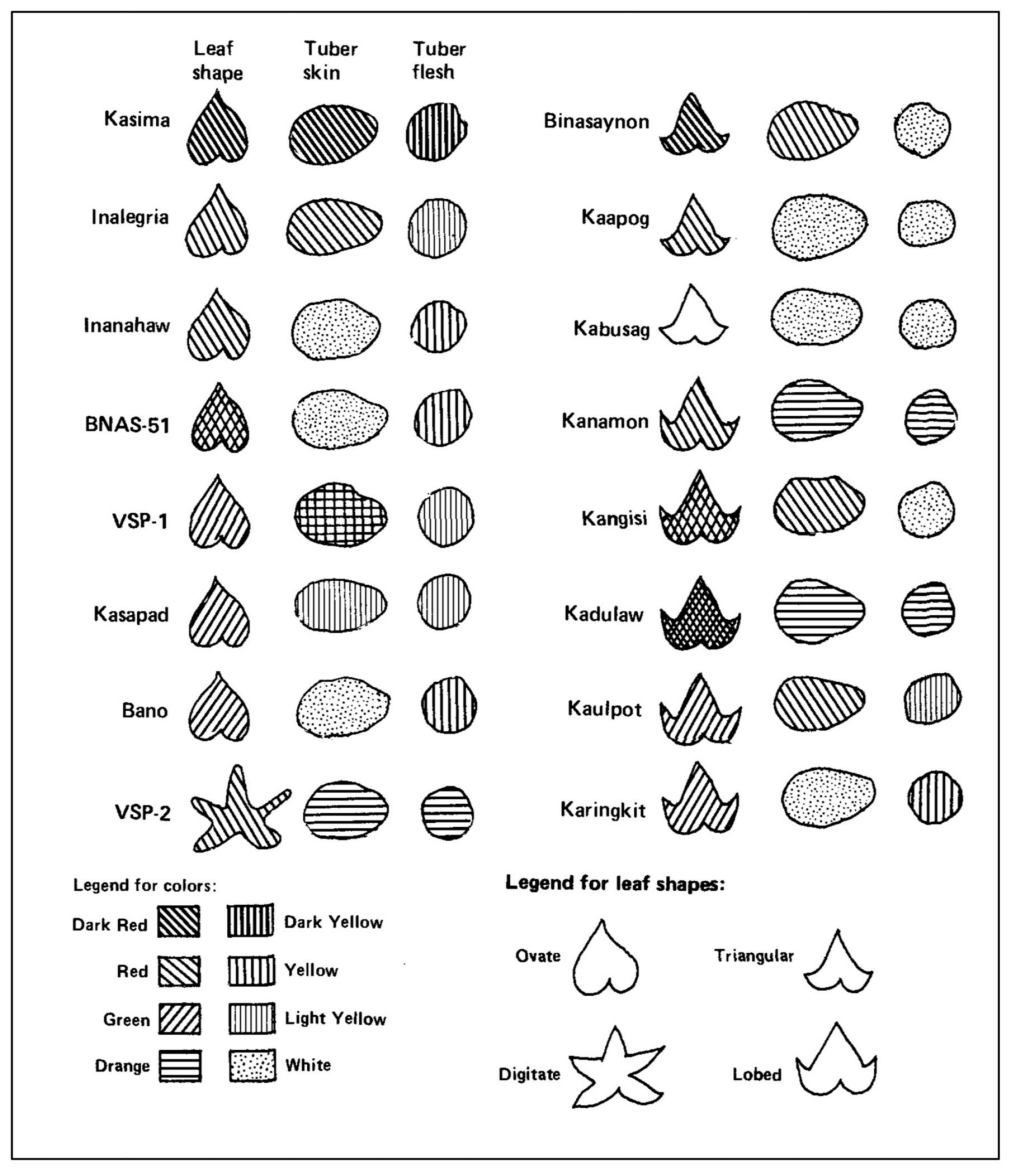
A diagram indicating the descriptions used by Filipino farmers in distinguishing different varieties of sweet potato. From C. Lightfoot, R. de Pedro, Jr., and F. Saladaga, “Screening of Sweet Potato Cultivars by Subsistence Farmers: Implications for Breeding,” in Kenneth T. Mackay, Manuel K. Palomar, and Rolinda T. Sanico, eds., *Sweet Potato Research and Development for Small Farmers* (Laguna, Philippines: SEAMEO-SEARCA, 1989), 48. Republished by permission of SEAMEO-SEARCA.

UPWARD-affiliated researchers implemented this vision in various ways, drawing on frameworks and methods shared across the growing domain of farmer-centered research. In one project, an agronomist from Cornell University and two researchers from the Visayas State College of Agriculture, Philippines “monitor[ed] indigenous research”—that is, Filipino farmers’ knowledge and experimentation—”in the farmers’ screening of sweet potato cultivators.” Their chief aim was to show “that these farmers’ needs cannot be mixed with commercial objectives” and to point the way towards breeding programs that would meet their needs.^77^ Using a mix of social science methods, from field surveys to participant observation to interviews, they sought to document “farmers’ experiments” and thereby elicit their preferences among varieties. As part of this research, the researchers documented the traits that farmers used to distinguish among the varieties in their fields. These included basic morphological features: leaf shape (ovate/heart-shaped, triangular/arrow head–shaped, lobed, and digitate/hand-shaped), leaf color (green, red, or red & green) and color of tuber skin and flesh (red, orange, yellow, and white) (See Figure 4.) They also attended to cultivation characteristics (including time to maturity, tuber size, pest and disease resistance, vining, and harvest period) and the qualities meaningful to farmers post-harvest, namely taste and storability.^78^ Demarcating varieties according to categories meaningful to farmers was, for this research team, a way of delivering information to breeders that would help them target smaller, poorer farming households—and therefore help international agricultural researchers “be more comfortable with our rhetoric of helping subsistence farmers.”^79^

It was one thing to collate this information and another entirely to make it accessible and meaningful beyond the social scientists who initially gathered it. How, for example, could information about the qualities that farmers sought be integrated into the databases that breeders increasingly relied on to access useful materials? Even in 1988, Huamán's first revised list of sweet potato crop descriptors had begun moving in this direction with its inclusion of the characteristics of tubers when boiled. But some researchers wanted to go much further. In 1992 a group of researchers including four researchers from Cenderawasih University in Indonesia, a CIP sweet potato breeder, and the UPWARD coordinator Prain generated an approach for “interdisciplinary collection” of sweet potato samples and “associated indigenous knowledge” intended to inform future breeding projects. Specifically, they sought means of “systematically incorporat[ing]” the “sophisticated knowledge of the [sweet potato] crop” held by farmers in Irian Jaya (today Papua province), Indonesia into conservation and evaluation programs. This meant devising approaches to documentation of farmers’ knowledge that would be compatible with existing databases and the descriptors used to populate these.^80^

To achieve these goals, the Cenderawasih University researchers and their collaborators assembled an interdisciplinary local team—two agronomists and two social scientists—that was tasked with conducting the field research. This research included collection of samples, surveys of and interviews with farmers, and elicitation of farmer knowledge using ethnobotanical methods; through these the team aimed to gather information on “the genetic makeup of the plant” (here its chiefly morphological features), “cultural knowledge about the plant,” and the “cultural, socioeconomic, and ecological characterization of the plant's environment.”^81^ Even considering all the challenges of obtaining farmer knowledge—having to begin with interviews with high status community members rather than “real experts,” having men speak for women, having interviewers’ “zeal to unearth local characterizations” result in farmers’ responding chiefly to please collectors, receiving conflicting accounts, experiencing many distractions and digressions in community meetings, and so on—it was even harder to move from the knowledge elicited to its systematic recording.^82^

As the researchers recounted, they gathered every evening after spending the day with farmers and in communities, to “discuss and brainstorm” over their individual notes and samples. They worked together to produce from these “a single daily record… structured around the topic guide”—an agreed list of desired information including ethnobotany, cultivation methods and local ecology specific to a sample, and broader patterns of farming and livelihoods—“and linked to the specimens via a collection number.”^83^ Some of this information was easy to then input into a database that could track the samples and knowledge associated with these, but not all. The researchers worried about “textual or discursive information.” Some could be rendered digital (and therefore input-able) by following the existing crop descriptor guidelines, but researchers worried that recording a farmer’s description of “blue-black” as “purple” (the nearest equivalent) missed the whole point of the ethnobotanical enterprise, which sought chiefly to record *farmers’* meaningful categories. Even more concerning were the cases where farmers’ knowledge was complex, as in the following example:

The practice of giving part cooked and part raw roots of certain cultivars to pigs and the reasons for doing this, … which were described by one farmer, were either not recorded, or were recorded as “cultivar x: use = pigfeed,” a formulation that is readily included in a matrix data base but which loses most of the nondigital information in the original statement.^84^

Finally, the natural scientists on the team saw some information as so superfluous to their potential future work that they insisted it be disregarded altogether, with no effort to codify and integrate it into the database. As the summary report noted, “ritual data” concerning “ritual practices associated with crops and farming” were “negotiated out of existence” in the process of interdisciplinary coordination.^85^

The group that sought and processed farmer knowledge in Irian Jaya (Papau) proposed two initial strategies for resolving the challenges of inputting complex cultural information into genebank databases. One was to better train researchers in making precise summaries of local knowledge. The second was to devise new approaches to data storage that would improve upon the standard approach—common in the preparation of descriptor lists and accompanying databases—of providing a “memo” field for characteristics where difficult to quantify information was thought likely to arise.^86^ In other words, they envisioned both better disciplining of researchers to systematic modes of description and changes to the functioning of databases. Their proposed solutions to the problem of diversity in sweet potato cultivation recognized that expanded descriptor lists and researcher training would not be sufficient to truly accommodate diverse knowledges: some basic elements of the dominant data infrastructure might need to change as well.

Other proposals for securing the cultural knowledge associated with farmers’ varieties tackled the limitations of standard crop and genebank databases head on. The anthropologist Virginia Nazarea, who served as the assistant coordinator of UPWARD, led a program of local knowledge collection and conservation of sweet potato in Bukidnon, Philippines in 1990 and 1991. Nazarea, born in the Philippines, had studied the decision making among Filipino rice cultivators for her doctoral research at the University of Kentucky in 1987. This ethnographic work reflected the priorities of farming systems research, interpreting cultivation patterns at the level of individual farmer, household, and local community.^87^ In her research conducted with UPWARD, Nazarea sought a “thorough documentation of cultural practices associated with traditional crop varieties, starting with sweet potato” following a protocol she dubbed “memory banking.”^88^ The resulting “repositories of farmers’ traditional knowledge” would provide a cultural complement to the genetic materials stored in genebank repositories and, according to Nazarea, “be a useful antidote to the posture, much resented by local farmers, of [technical experts] pouring agricultural ‘know-how’ into what are presumed to be empty vessels.”^89^

**Figure 5:**
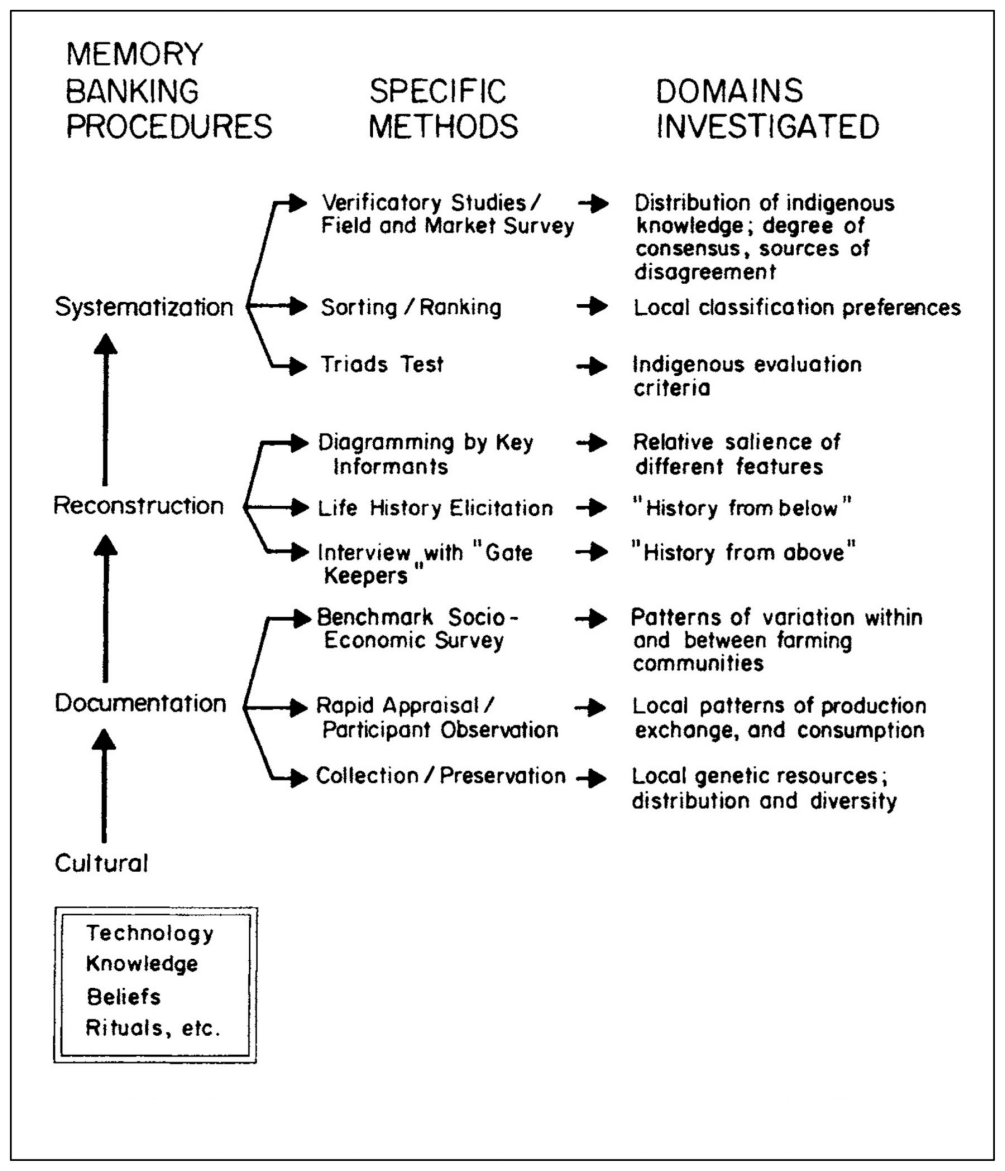
The memory banking procedures elaborated by Virginia Nazarea used a wide range of methods to capture an equally wide range of knowledge and experience. From Virginia D. Nazarea, *Cultural Memory and Biodiversity* (Tucson: University of Arizona Press, 1998), 18. Republished by permission of the University of Arizona Press.

Over more than a year, Nazarea employed a range of methods—interviews, surveys, participant observation, life histories, collaborative farming, community conservation, and more—to produce a memory bank of fifty-plus farmers’ varieties of sweet potatoes. The documentation included farmers’ and scientists’ characterizations of each variety, farmers’ and scientists’ drawings, farmers’ criteria for evaluating sweet potatoes, compilations of beliefs and practices linked to sweet potatoes, audio recordings and transcripts of farmers’ life histories, and agricultural calendars revealing (for example) the timing of planting and harvesting.^90^ (See Figure 5.) Tellingly, one of the other UPWARD associates, the anthropologist Jürg Schneider, characterized Nazarea’s memory banking method of describing samples as “the maximal approach to documentation of indigenous knowledge.”^91^

Schneider also observed some of the challenges that inhered in the memory banking method. In his assessment, these included chiefly the “absence of uniformity in local knowledge” and the “influence of research personalities.” With respect to the first, the variation encountered in the “description and criteria given for local varieties” by farmers placed onus on the memory banker to decide if and when these variations were meaningful. With respect to the second, concern arose about replicability. Would every researcher elicit the same information? If not, the content of the memory bank assembled by any individual researcher would be as unique to that researcher as to the community from which the knowledge was drawn, ultimately raising questions about the comparability of data from one memory bank to the next.^92^

These were precisely the circumstances that had spurred the development of crop descriptor lists: the desire to corral observations of diverse plants made by diverse researchers into tidy, consistent categories easily entered into a database and to expunge as much as possible the subjectivity of the researcher. The pursuit of a mode of documentation for genebank collections that was maximally inclusive in terms of the range and form of description—as memory banking was and is—therefore diverged sharply from the technical project of crop description as imagined in preceding decades. Rather than insist on universalized language and disciplined observers as the solution to documenting diversity, it sought to accommodate many ways of seeing and knowing the world.

## The price of maximalism

That the maximal documentation of local knowledge would ultimately abandon the principles as well as the content of the maximal descriptor list should not come as a surprise. The ambitions of memory banking were to capture the diversity of local cultures and ways of being, not to standardize them. What’s more interesting is to observe the comparable drive towards inclusivity in both crop documentation enterprises. Responding to the demands of an international agricultural research community increasingly sensitive to the critiques of narrowness and exclusivity, scientists took pains to develop tools and approaches that would enable as wide as possible a range of participants, plants, and knowledges.

But maximalism ultimately produced its own exclusivity. The list of challenges to memory banking ought to also have highlighted its need for greater time and resources to be successful in comparison to other approaches. It was simply too labor-intensive and time consuming to be carried out except in isolated projects or by niche organizations.^93^ The same was true of the new maximalist approach to internationally agreed crop descriptor lists. Although the International Board for Plant Genetic Resources had confirmed its crop descriptor lists as allowing the “widest number of descriptors” in 1992, it soon reversed direction. Fantastically comprehensive descriptors proved cumbersome for breeders with fewer resources, potentially exacerbating existing disparities.^94^ Comprehensive descriptors were not abandoned, but instead accompanied by a reduced, general list of “minimal,” “highly discriminating” descriptors that—it was claimed—could be applied across species and locations. This new format, first trialed with barley in 1994, was thought to make descriptors more user friendly—one hardly needs add—*again*.^95^

Removed from the immediate exigencies of documenting and managing thousands of diverse plant samples *ex situ*, *in situ*, and *in silico* on behalf of an international research community, the vision of creating a vocabulary or mode of documentation that could accommodate all plants and all people and all environments may seem hopelessly optimistic. Yet as I have shown, these efforts took shape as institutions and individuals endeavored to respond to critiques of international agricultural research, not only by adopting new objects of study such as sweet potatoes and farm systems but also by attempting to change practices as fundamental as the description of roots, leaves, fruits, and flowers. That the world proved too diverse and too large to be successfully encompassed in new systems of description should not distract from the important fact of researchers having grappled with the need to accommodate diversity in meaningful ways.

To date, critical histories of international agricultural research have focused mostly on the failures of top-down agricultural interventions. They have—rightly—highlighted the limitations of attempting to solve the food and farming problems of diverse communities and ecologies through blanket solutions. But the institutions credited with the Green Revolution were not insensitive to the critiques that emerged close on the heels of that initial, apparent agricultural transformation. If historians are to grapple still more fully with the legacies of the Green Revolution, it is essential they also chart the trajectories of research efforts that, like efforts to integrate the study of sweet potatoes into international agricultural research systems, responded to the criticisms of top-down agricultural development and embraced alternative approaches and outcomes.

## Footnotes

1 Villareal, “Sweet Potato in the Tropics,” 4. His characterization reflected the perspective of international research scientists and institutions and not necessarily that of farmers and families who depended on sweet potatoes for survival.

2 An early and influential body of research critical of the Green Revolution emerged from a programme of the United Nations Research Institute for Social Development; see, e.g., Griffin, *Green Revolution*; Hewitt de Alcántara, *Modernizing Mexican Agriculture*; Pearse, *Seeds of Plenty, Seeds of Want*. Other key early critical assessments include Frankel, *India's Green Revolution*; Cleaver, *Origins of the Green Revolution*. A more comprehensive bibliography of the early literature can be found in Oasa, “International Rice Research Institute and the Green Revolution,” 55, n. 2.

3 Glaeser, *Green Revolution Revisited*; an overview of some of these shifts is found in Özgediz, *CGIAR at 40*. This aspect of the history of the Green Revolution has yet to be subjected to sustained historical attention. Some early responses to the perceived shortcomings of the research and technology development programs associated with the Green Revolution are examined in contributions to Curry and Lorek, *Research as Development*, especially chapters by Lucas Mueller (on “later stage development problems”), Prakash Kumar (on research for non-irrigated agriculture) and Harro Maat (on upland rice cultivation programs in Africa). See also van der Burg, “‘Change in the Making.’” For actors’ accounts of efforts to diversify the targets of research, see Moock and Rhoades, *Diversity, Farmer Knowledge and Sustainability*. An account that considers 1980s responses to the perceived failures of international economic development programs more generally is Meyerowitz, *War on Global Poverty*.

4 Villareal, “Sweet Potato in the Tropics – Progress and Problems.”

5 To be clear, there were many researchers with significant expertise on sweet potato, especially within Asian agricultural institutions, and an expanding transnational network of sweet potato researchers (see, e.g., Villareal and Griggs, *Sweet Potato*). My focus here is on institutional developments that attempted to forge new, explicitly international programs within CGIAR and its affiliated international agricultural research centers.

6 See discussions in Groenfeldt and Moock, *Social Science Perspectives on Managing Agricultural Technology*; Cernea and Kassam, *Researching the Culture in Agri-Culture*; Price and Palis, “Bringing Farmer Knowledge and Learning into Agricultural Research.” This history is discussed in Curry, *Endangered Maize*, ch. 7.

7 Useful entry points into to this historiography include Kumar et al., “Roundtable”; Lorek, “Green Revolution in Latin America”; and Stone, “Commentary.” For examples see, Gutiérrez Núñez, “Revolución Verde en los Suelos Agrícolas de México”; Kumar, “‘Modernization’ and Agrarian Development in India”; Lin, “Sowing Seeds and Knowledge”; Lorek, “Puerto Rican Connection”; Méndez Rojas, “Maize and the Green Revolution”; Nehring, “Brazilian Green Revolution”; Picado Umaña, “Technology, Geopolitics, and Institutions”; Saha, “Food for Soil, Food for People”; Soto Laveaga, “Beyond Borlaug’s Shadow”; Subramanian, “Revisiting the Green Revolution.”

8 On the history of crop descriptor development within CGIAR, see Curry and Leonelli, “Describing Crops in the CGIAR Era”; Gotor et al., “The Scientific Information Activity of Bioversity International.”

9 On earlier institutional developments in international agricultural research, see Byerlee and Lynam, “Development of the International Center Model for Agricultural Research.”

10 See, e.g., Anderson, “Origins of the International Rice Research Institute”; Perkins, *Geopolitics and the Green Revolution*; Cullather, “Miracles of Modernization”; Cullather, *Hungry World*. The main institutional accounts are Byerlee, *Birth of CIMMYT*; Chandler, *Adventure in Applied Science*.

11 For different assessments of the Green Revolution, see Evenson and Gollin, “Assessing the Impact of the Green Revolution”; Harwood, *Europe's Green Revolution and Others Since*; Pingali, “Green Revolution”; Patel, “The Long Green Revolution.”

12 For evidence, see emphasis on building research institutions in contributions to *Agricultural Development*, an account of a high-level meeting of donors and aid agencies that ultimately led to the establishment of CGIAR.

13 These were the International Center for Tropical Agriculture (CIAT, *Centro International de Agricultura Tropical*) in Cali, Colombia, and the International Institute for Tropical Agriculture (IITA) in Ibadan, Nigeria.

14 On the institutional development of CGIAR, see Özgediz, *CGIAR at 40*.

15 AVRDC, *Annual Report for 1973–74*, 6. See Chu, “With and Against the Grain”; Lin, “Sowing Seeds and Knowledge.” I am grateful to Leo Chu for introducing me to the geopolitical history of AVRDC and the trajectory of vegetable research carried out there.

16 AVRDC, *Annual Report for 1973–74*.

17 Chandler, “Asian Vegetable Research and Development Center.”

18 Robert Chandler to Peter Oram, 30 May 1972, included in materials for the Fourth Meeting of the CGIAR Technical Advisory Committee, 2–4 August 1972, https://hdl.handle.net/10947/1199.

19 TAC Secretariat, *Report of the Fourth Meeting of the Technical Advisory Committee*, 5.

20 Chu, “With and Against the Grain.” See also “Chu, “Harvesting Diversity.”

21 It would also conduct collaborative research on cassava. TAC Secretariat, *Report of the Second Meeting of the Technical Advisory Committee*, 13–14.

22 Hahn, “Progress on Root and Tuber Improvement at IITA,” 56–57.

23 CGIAR Secretariat, *Summary of Proceedings of the Second Meeting of CGIAR*, 3.

24 Sawyer, “El Centro Internacional de la Papa.”

25 Sawyer, “El Centro Internacional de la Papa,” 416.

26 Sawyer, “The International Potato Center,” 4.

27 See discussion in TAC Secretariat, “Priorities for International Support to Agricultural Research in Developing Countries,” esp. 8–9.

28 Harwood, “Was the Green Revolution Intended to Maximise Food Production?"

29 FAO, “FAO ‘Overview,’” 6.

30 Reeves, *International Institute of Tropical Agriculture, 1967–1997*.

31 Sawyer, “Opening Sweet Potato Planning Conference," 1.

32 Beintema and Echeverría, “Evolution of CGIAR Funding.”

33 TAC Secretariat, “TAC 30 Discussion Paper,” 15–16, 18.

34 TAC Secretariat, “Strategic Issues and Priorities,” 11.

35 TAC Secretariat, *Report of the Thirty Seventh Meeting of the Technical Advisory Committee*, 29–31.

36 As noted, IITA in Nigeria had the CGIAR “global mandate” for sweet potatoes. TAC Secretariat, *Report of the Thirty Seventh Meeting of the Technical Advisory Committee*, 29–30.

37 Initially, responsibility was assigned to IITA in Nigeria and two US Department of Agriculture institutions; the AVRDC was assigned responsibility for maintaining Asian and Pacific region collections in 1984. See IBPGR, *Genetic Resources of Sweet Potato*, 2.; Fernandez, Lu, and Huang, “Status of Sweet Potato Collections Maintained at AVRDC,” 129.

38 Fernandez, Lu, and Huang, “Status of Sweet Potato Collections Maintained at AVRDC,” 132.

39 On the challenges of maintaining virus free potato stocks, see Joaquim, “Agricultural Virology in Mid-Twentieth-Century Britain,” PhD dissertation, University of Cambridge, in preparation.

40 Sawyer, “Opening Sweet Potato Planning Conference.”

41 Huamán, “Current Status on the Maintenance of Sweet Potato Genetic Resources at CIP,” 105–106.

42 Bioversity International, “Descriptors.”

43 Curry and Leonelli, “Describing Crops in the CGIAR Era”; Gotor et al., “The Scientific Information Activity of Bioversity International.” On the political economy of genebanking and practical experiences of genebank curation, see Peres, “Saving the Gene Pool”; Xan Chacko, “Moving, Making, and Saving Botanic Futures.”

44 IBPGR, *Genetic Resources of Sweet Potato*. Absent members of the committee included scientists from Brazil, Cuba, and Japan.

45 IBPGR, *Genetic Resources of Sweet Potato*.

46 For a detailed account of Huamán’s education and research career, see Huamán Cuevas, “Perú, cuna de las papas más exquisitas.”

47 Huamán, *Sweetpotato Germplasm Management* (Ipomoea batatas).

48 Huamán, “Current Status on the Maintenance of Sweet Potato Genetic Resources at CIP,” 117.

49 Huamán, “Current Status on the Maintenance of Sweet Potato Genetic Resources at CIP,”115. Huamán also relied on protein and enzyme analyses and other biochemical methods; see Huamán, *Morphologic Identification of Duplicates in Collections of* Ipomoea batatas.

50 Huamán, “Current Status on the Maintenance of Sweet Potato Genetic Resources at CIP,” 117.

51 Fernandez, Lu, and Huang, “Status of Sweet Potato Germplasm Maintained at AVDRC,” 133, 135.

52 Tagaki, “Sweet Potato Collections in Papua New Guinea,” 149.

53 Huamán, “Current Status on the Maintenance of Sweet Potato Genetic Resources at CIP,” 117.

54 Huamán, “Descriptors for the Characterization and Evaluation of Sweet Potato Genetic Resources,” 331–332.

55 Huamán, “Descriptors for the Characterization and Evaluation of Sweet Potato Genetic Resources,” 340–341, 353–354.

56 IBPGR, *Genetic Resources of Sweet Potato*, 23.

57 Huamán, “Descriptors for the Characterization and Evaluation of Sweet Potato Genetic Resources,” 342–344.

58 Huamán, *Morphologic Identification of Duplicates in Collections of* Ipomoea batatas, 27. See also Huamán, *Sweetpotato Germplasm Management* (Ipomoea batatas), sect. 1.3.

59 J. M. M. Engels, “Data Management within a Sweet Potato Genetic Resources Network,” 141, 143.

60 CIP, AVDRC, and IBPGR, *Descriptors for Sweet Potato*, preface.

61 TAC Secretariat, *Report of the TAC Quinquennial Review of the International Board for Plant Genetic Resources (IBPGR)*, 22.

62 IBPGR, *Annual Report 1992*, 37.

63 CIP, AVDRC, and IBPGR, *Descriptors for Sweet Potato*.

64 On early critiques of the Green Revolution, see references in fn. 2.

65 Özgediz, *CGIAR at 40*, 19.

66 Collinson, “Introduction,” 2.

67 See discussion in Curry, *Endangered Maize*, ch. 7.

68 Warren, “Using Indigenous Knowledge in Agricultural Development.”

69 Rhoades and Booth, “Farmer-Back-to-Farmer.”

70 Thiele, van de Fliert and Campilan, “What Happened to Participatory Research at the International Potato Center?” 430–1.

71 Nazarea, “Potato Eyes,” 4.

72 Thiele, van de Fliert and Campilan, “What Happened to Participatory Research at the International Potato Center?” 434.

73 Robert E. Rhoades, interview with Jan van Willigen, 7 October 2002, https://www.appliedanthro.org/application/files/3615/5924/0637/oral-history-Rhoades-VanWilligen.pdf (accessed 2 September 2022). See also Thiele, van de Fliert and Campilan,“What Happened to Participatory Research at the International Potato Center?” 434.

74 MacKay, “[Sweet] Potato, Small Farmers, and Need for Cooperative Research,” 38–9.

75 MacKay, “[Sweet] Potato, Small Farmers, and Need for Cooperative Research,” 34.

76 Prain, “Preface: A Brief History of Upward,” iv, vi, vii.

77 Lightfoot, de Pedro, and Saladaga, “Screening of Sweet Potato Cultivars by Subsistence Farmers,” 44.

78 Lightfoot, de Pedro, and Saladaga, “Screening of Sweet Potato Cultivars by Subsistence Farmers,” 49.

79 Lightfoot, de Pedro, and Saladaga, “Screening of Sweet Potato Cultivars by Subsistence Farmers,” 54.

80 Sawor et al., “Methods for Interdisciplinary Collection of *Ipomoea batatas* Germplasm,” 162.

81 Sawor et al., “Methods for Interdisciplinary Collection of *Ipomoea batatas* Germplasm,” 164.

82 Sawor et al., “Methods for Interdisciplinary Collection of *Ipomoea batatas* Germplasm,” 167.

83 Sawor et al., “Methods for Interdisciplinary Collection of *Ipomoea batatas* Germplasm,” 168.

84 Sawor et al., “Methods for Interdisciplinary Collection of *Ipomoea batatas* Germplasm,” 169.

85 Sawor et al., “Methods for Interdisciplinary Collection of *Ipomoea batatas* Germplasm,” 170.

86 Sawor et al., “Methods for Interdisciplinary Collection of *Ipomoea batatas* Germplasm,” 169.

87 Nazarea-Sandoval, *Local Knowledge and Agricultural Decision Making in the Philippines*.Nazarea was married to Robert Rhoades and the pair continued collaborative (and individual) work in agricultural anthropology and participatory research after their time at UPWARD. See Nazarea, “Potato Eyes.”

88 Nazarea, *Cultural Memory and Biodiversity*, 12; see also Sandoval, “Memory Banking: The Conservation of Cultural and Genetic Diversity in Sweetpotato Production”; Nazarea-Sandoval, “Memory Banking of Indigenous Technologies of Local Farmers Associated with Traditional Crop Varieties.”

89 Sandoval, “Memory Banking,” 25–6.

90 Nazarea, *Cultural Memory and Biodiversity*, ch. 2.

91 Schneider, “UPWARD and Rootcrop Genetic Resources,” 55.

92 Schneider, “UPWARD and Rootcrop Genetic Resources,” 55–56.

93 For further examples of memory banking (including two directly connected to Nazarea) see Rhoades, “Saving the Southern Legacy”; van Dooren, “Banking Seed”; Veteto, “The History and Survival of Traditional Heirloom Vegetable Varieties in the Southern Appalachian Mountains of Western North Carolina”; Vogl-Lukasser, Vogl and Blauensteiner, “Link between Protected Areas and Agrobiodiversity Conservation.”

94 Gotor et al., “Scientific Information Activity of Bioversity International.”

95 IPGRI, *Annual Report 1993*.
